# A Systematic Review of Prolonged SARS‐CoV‐2 Shedding in Immunocompromised Persons

**DOI:** 10.1111/irv.70121

**Published:** 2025-05-20

**Authors:** Rebecca C. Christofferson, Jennifer E. Giovanni, Emily H. Koumans, Muyiwa Ategbole, Samantha D. Clark, Shana Godfred‐Cato, Manoj P. Menon, Inka Sastalla, Beth K. Schweitzer, Timothy M. Uyeki

**Affiliations:** ^1^ Louisiana State University School of Veterinary Medicine Baton Rouge Louisiana USA; ^2^ Centers for Disease Control and Prevention Atlanta Georgia USA; ^3^ Fred Hutchinson Cancer Center Seattle Seattle Washington USA; ^4^ Office of Genomics and Advanced Technologies National Institute of Allergy and Infectious Diseases National Institutes of Health Bethesda Maryland USA

**Keywords:** COVID‐19, immunocompromised, SARS‐CoV‐2

## Abstract

**Background:**

Although reports have documented prolonged SARS‐CoV‐2 RNA detection in immunocompromised patients, few studies have systematically analyzed data on duration of SARS‐CoV‐2 in respiratory specimens of immunocompromised patients.

**Methods:**

A systematic review was undertaken to describe SARS‐CoV‐2 RNA and infectious virus detection in immunocompromised patients from published data between January 1, 2020 and July 1, 2022. Patients were included if there was ≥ 1 positive SARS‐CoV‐2 RNA result in respiratory specimens collected > 20 days since symptom onset or first positive SARS‐CoV‐2 RT‐PCR result.

**Results:**

Of the 183 patients, 175 were symptomatic with 83 (47.4%) that experienced intermittent relapsing symptoms, while pneumonia was reported in 122 (66.7%). Immunocompromising conditions represented were hematologic malignancy treatment (89, 48.6%), solid organ transplant (47, 25.7%), autoimmune disease treatment (14, 7.7%), solid tumor treatment (3, 1.6%), HIV infection (15, 8.2%), and primary immunodeficiency (15, 8.2%). Median duration from the first to the last positive SARS‐CoV‐2 RT‐PCR result was 56 days in upper respiratory and 60 days in lower respiratory tract specimens. Significant differences in median duration of SARS‐CoV‐2 RNA detection were observed between patients with and without pneumonia and for patients with hematologic malignancies compared to solid organ transplant patients. Among patients with viral culture performed, median duration of replication‐competent SARS‐CoV‐2 was 60.5 days from symptom onset (maximum 238 days) and 59 days from first RT‐PCR positive result (maximum 268 days).

**Conclusions:**

Immunocompromised persons can have replication‐competent SARS‐CoV‐2 in respiratory tissues for months, including while asymptomatic. Serial SARS‐CoV‐2 testing can inform the duration of isolation for immunocompromised patients with SARS‐CoV‐2 infection.

## 1 Introduction

The duration of severe acute respiratory syndrome coronavirus 2 (SARS‐CoV‐2) in the respiratory tract of infected persons is generally well understood [1, 2], but few studies have systematically reviewed SARS‐CoV‐2 RNA persistence in the respiratory tracts of immunocompromised patients. Although case reports have documented SARS‐CoV‐2 RNA in immunocompromised patients for weeks to over a year [3, 4, 5], the implications of persistent detection of SARS‐CoV‐2 are not fully understood. Viral evolution and immune evasion have been described among immunocompromised patients with persistent infection [5, 6, 7, 8, 9]. Understanding factors that may influence persistence of SARS‐CoV‐2 and the period during which immunocompromised patients are infectious is important to guide public health measures to limit transmission and inform clinical management.

During the COVID‐19 pandemic, the Centers for Disease Control and Prevention (CDC) recommended isolation of people with COVID‐19 who are moderately or severely immunocompromised for at least 20 days, with serial SARS‐CoV‐2 testing and consultation with an infectious disease specialist to help determine the duration of isolation and precautions. Because these recommendations were made early in the COVID‐19 pandemic when there were limited data available on the duration of SARS‐CoV‐2 persistence among immunocompromised persons, we performed a systematic review of published reports on SARS‐CoV‐2 RNA persistence exceeding 20 days in the respiratory tract of immunocompromised patients to assess whether the guidance was still applicable. We describe the clinical characteristics, disease course, and outcomes associated with prolonged detection of SARS‐CoV‐2 RNA and infectious virus in the respiratory tracts of immunocompromised patients.

## 2 Materials and Methods

### 2.1 Search Strategy and Selection Criteria

An initial search was conducted to identify publications describing immunocompromised patients with persistent detection of SARS‐CoV‐2 RNA in respiratory specimens. Immunocompromised patients were defined as those with moderate or severe immunocompromising conditions or receipt of immunosuppressive medications [10]. A systematic literature search was performed using key words identified from an initial retrieval of 120 publications. Additionally, the reference lists of review articles were screened for potential relevant primary studies, and the reference lists of included primary studies were further searched for studies for potential inclusion. Publications were retrieved from PubMed, Embase, Global Health, Cochrane Library, CINAHL, and Scopus databases from January 1, 2020 through July 1, 2022. Additional references were identified through reference lists and Google Scholar. The search strategy is available in the Supporting Information S1.

### 2.2 Inclusion Criteria

This review included immunocompromised patients with reported SARS‐CoV‐2 RNA detection by RT‐PCR from respiratory specimens (nasopharyngeal, throat, sputum, bronchoalveolar lavage, and endotracheal tube) with at least one positive result > 20 days since the first onset of symptoms or > 20 days after an initial positive result for asymptomatic patients. Studies were included if sufficient patient level information was available on immunocompromising diagnosis and SARS‐CoV‐2 reverse transcription‐polymerase chain reaction (RT‐PCR) testing results. There were no age restrictions. Studies with at least one individual who was immunocompromised were included.

### 2.3 Exclusion Criteria

Excluded publications were those in languages other than English, reviews without primary data, reports presented solely as abstracts at scientific conferences, preprints, those that reported data in a poster or abstract, or only reported results from testing of postmortem or nonrespiratory specimens. Publications where reinfection was established with viral genomic sequencing were excluded.

### 2.4 Screening

Eight reviewers (RCC, SDC, MA, SGC, JG, MM, BS, TU) independently scanned the abstract, title, or both, to identify eligible publications for full‐text review, and then performed full‐text review to assess inclusion and exclusion criteria.

### 2.5 Selection and Data Extraction

For included studies, nine reviewers (MA, RCC, SDC, SGC, JG, EK, MM, BS, and TU) independently abstracted data into an Excel spreadsheet to capture demographic, clinical, and laboratory information. Patients were included based on the availability of viral RNA persistence data (qualitative) that met the > 20‐day SARS‐CoV‐2 RNA positive threshold. Immunocompromising conditions were classified into six categories: immunosuppressive therapy for autoimmune disease (A), hematologic malignancy (H), human immunodeficiency virus infection (HIV), primary immunodeficiency (PI), solid organ transplant recipient (T), or solid tumor malignancy (ST). Hematologic malignancy patients were subclassified into (a) bone marrow transplant (BMT) recipient, (b) chimeric antigen receptor T‐cell (CAR‐T) therapy recipient, or (c) non‐BMT/CAR‐T recipient. Two reviewers (SC and RC) independently reviewed all included patients and rechecked the abstracted data for accuracy in consultation with one reviewer (TU) for discrepancies.

### 2.6 Risk of Bias Assessment

Risk of bias assessment was carried out using the appropriate tool from the Joanna Briggs Institute (JBI) (i.e., either for case studies, case series, or cohort studies) [11, 12, 13]. As suggested by the appraisal tools, each included publication was subjected to appraisal by two authors (see Supporting Information Methodology for details). To assess confidence in our main effect of interest (long‐term duration of SARS‐CoV‐2 RNA detection in respiratory specimens), the following system was employed. A patient was classified as having prolonged SARS‐CoV‐2 RNA detection if (a) there was viral sequencing evidence inconsistent with SARS‐CoV‐2 reinfection between the first and last respiratory specimens that tested positive by RT‐PCR for SARS‐CoV‐2; or (b) the maximum time between first and last respiratory specimens that were positive for SARS‐CoV‐2 by RT‐PCR was < 90 days without a negative result between positive results. A patient was classified as having probable prolonged SARS‐CoV‐2 RNA detection if the maximum time between first and last respiratory specimens that were positive for SARS‐CoV‐2 by RT‐PCR was ≥ 90 days without viral sequencing data available.

### 2.7 Data and Analyses

Data were abstracted on age, sex, immunocompromise diagnosis, presence or absence of symptoms and relapsing symptoms, hospitalization status, and the results of all RT‐PCR testing per included patient and viral culture (where available). Upper respiratory tract (U) specimens were defined as nasal, nasopharyngeal (NP), oropharyngeal (OP), or saliva. Lower (L) respiratory specimens were defined as bronchoalveolar lavage (BAL), bronchial secretion (BS), endotracheal aspirate, sputum, or tracheal biopsy. There were four patients where the respiratory specimen could not be inferred because the authors stated that “confirmation of SARS‐CoV‐2 infection was done by qualitative real‐time RT‐PCR assay of nasopharyngeal swabs, sputum, or lower respiratory tract aspirates [14].” Patients were classified with pneumonia if radiographic evidence of pulmonary infiltrates, opacities, or consolidation was included in the publication. The duration of viral RNA persistence was defined as the reported duration in days from the time of symptom onset to the last positive RT‐PCR result or time between the first and last positive RT‐PCR test results. The duration of infectious virus was defined as the last positive viral culture from illness onset.

For descriptive analyses, qualitative (i.e., positive/negative) RT‐PCR results from patients were used to determine the maximum duration of SARS‐CoV‐2 RNA detection in the upper versus lower respiratory tracts. Ct values without date of specimen collection were excluded. Comparisons of duration of RNA results across demographics variables and immunocompromising conditions were conducted using the Kruskal–Wallis nonparametric analysis of variance, with significance assessed at the α = 0.05 level. Where appropriate, post hoc pairwise comparisons were conducted using a Bonferroni adjustment.

This systematic review was conducted in accordance with the Preferred Reporting Items for Systematic Reviews and Meta‐Analyses (PRISMA) statement [15], and registered with the International Prospective Register of Systematic Reviews (PROSPERO; CRD42023408783). All analyses and visualizations were performed using R/R Studio (versions 4.3.2 and 2023.09.1 Build 494, respectively).

## 3 Results

**FIGURE 1:**
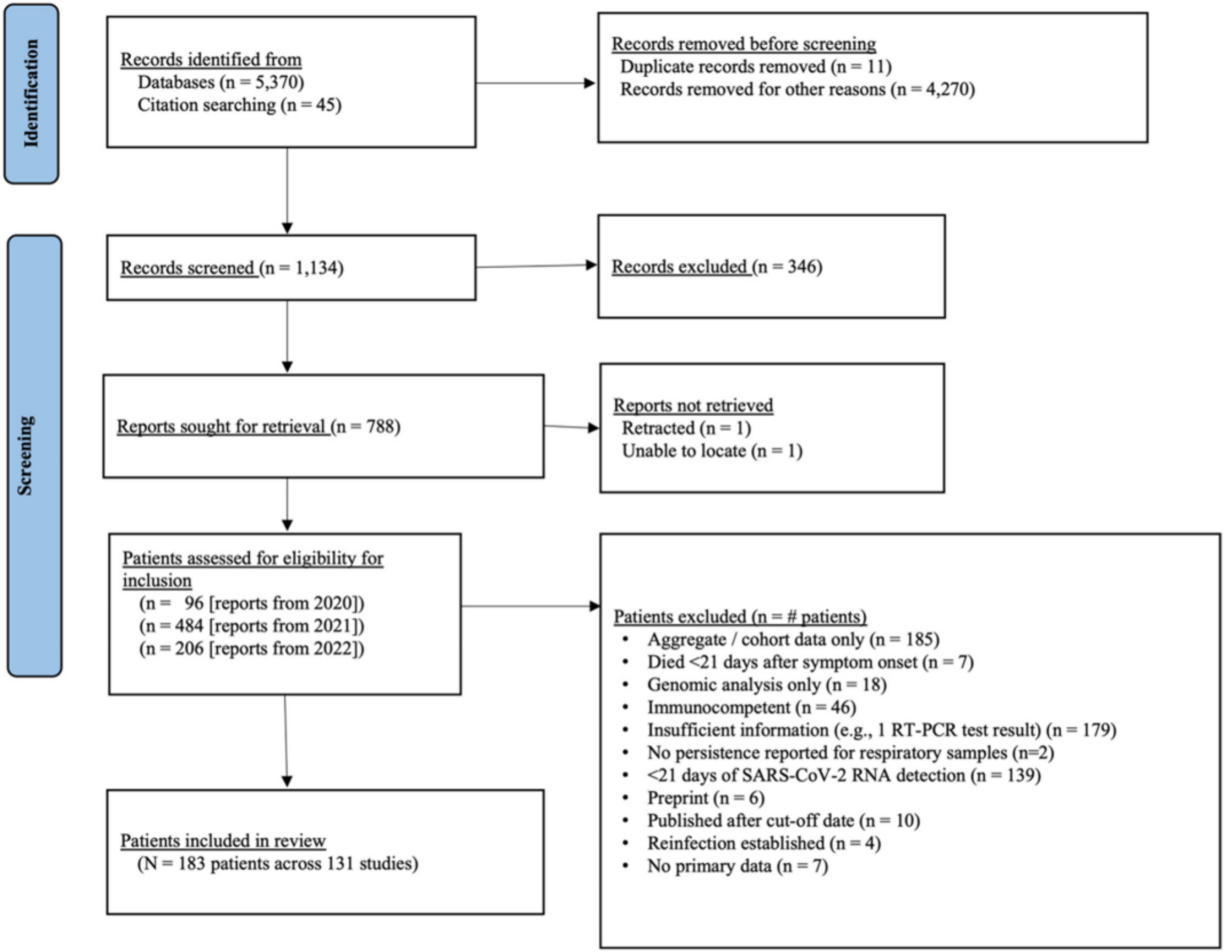
PRISMA flow chart of studies screened for inclusion.

A total of 5,370 publications from January 1, 2020, through July 1, 2022, were identified through literature review. After screening and full‐text review of 786 studies eligible for inclusion, 139 publications from 32 countries with a total of 197 patients were identified that met inclusion criteria. Following a Risk of Bias Assessment (Supporting Information S1), an additional 14 patients from 9 publications were excluded. The final dataset included 131 publications from 31 countries with 183 patients (Figure 1). Of the included studies, 103 (78.6%) were case reports, 22 (16.8%) were case series, and 6 (4.6%) were cohort studies. In 162 (88.5%) of patients, prolonged SARS‐CoV‐2 RNA detection was confirmed based on our defined criteria, while 21 (13.0%) patients were classified with probable prolonged SARS‐CoV‐2 RNA detection. Of those classified with confirmed prolonged viral RNA detection, 115 patients had SARS‐CoV‐2 RT‐PCR positive results within 90‐days (71.0%) and 47 (29.0%) had viral sequencing evidence of prolonged SARS‐CoV‐2 RNA detection (Figure S1).

### 3.1 Demographics

Of the 183 patients included in the analysis (Table 1), 172 (94.0%) had age reported. The median age was 53 years, range 2.5 months to 76 years (interquartile range [IQR] 37, 63.5 years). Information on sex was available for 169 (92.4%) patients; of those, 91 (44.3%) were male, 78 (46.2%) were female (Table 1).

**TABLE 1:** Characteristics of immunocompromised patients with detection of SARS‐CoV‐2 RNA included in systematic review.

Characteristic	Number of patients (*N* = 183)
Age class	
< 18 years	13
18–49 years	63
50–64 years	63
*≥* 65 years	39
Unknown	5
Sex	
Female	78
Male	91
Unknown	14
Symptomatic	
Yes	175
No	8
Relapsing symptoms (symptomatic patients *n* = 175)	
Yes	83
No	71
Unknown	29
Hospitalization	
Yes	173
> 1 admission	69
No	6
Unknown	4
Pneumonia diagnosis	
Yes	122
No	20
No data provided	41
Outcome	
Survived	145
Deceased	26
Still hospitalized at time of manuscript prep	2
Unknown	10
Immunodeficiency	
Autoimmune disease treatment	14
Hematologic malignancy treatment	89
Bone marrow transplant	10
CAR‐T therapy	5
Unspecified	74
HIV, by CD4+ cell count	15
< 50	8
50–200	2
> 200	5
Primary immunodeficiency	15
Solid tumor malignancy treatment (lung)	3
Solid organ transplant treatment	47
Heart	10
Kidney	26
Liver	6
Lung	5

### 3.2 Clinical Characteristics

Among the 183 included patients, 175 were symptomatic (95.6%), and 8 were asymptomatic (4.4%) (Table 1). Of the symptomatic patients, 83 (47.4%) experienced intermittent relapsing symptoms.

Information on hospitalization was reported for 180 (97.8%) of patients; among the 173 (94.5%) patients who were hospitalized, 69 (39.9%) had multiple hospital admissions. Pneumonia was reported in 122 (66.7%) of patients. Among 173 (94.5%) with outcome reported, 26 (15.0%) died, 145 (83.8%) survived, and 2 (1.2%) remained hospitalized at the time of the publications citing these patients.

### 3.3 Immunocompromising Diagnoses

Of all 183 patients, 89 (48.6%) had an underlying hematologic malignancy, and of those, 10 (11.2%) had received a bone marrow transplant, and 5 (6.2%) had received CAR‐T therapy (Table 1). Forty‐seven (25.7%) patients were solid organ transplant recipients, 14 (7.7%) had received immunosuppressive treatment for an autoimmune disease, and 3 (1.6%) patients had received treatment for solid tumor malignancies. Fifteen (8.2%) patients were diagnosed with HIV, and 15 (8.2%) had a primary immunodeficiency.

### 3.4 Detection of Viral RNA in Respiratory Specimens (*N* = 183 Patients)

In 159 (86.3%) patients, the same type of clinical specimen was reported for the first and last SARS‐CoV‐2 RT‐PCR positive results. The remaining patients had unmatched respiratory specimens from the first to the last RT‐PCR result (*n* = 20) or no data were available on the final specimen type (*n* = 4). Among patients with those 20 unmatched specimens, 5 (25.0%) matched in specimen source location (i.e., upper vs. lower respiratory tract). Thirteen (65.0%) had an initial positive result on an upper respiratory tract specimen and a last reported positive on a lower respiratory tract specimen and two (10.0%) had initial positives on lower respiratory tract specimens and last positives on upper respiratory tract specimens. In nine patients (4.9%), multiple specimens were reported at the last timepoint for a total of 192 total specimens representing the last positive SARS‐CoV‐2 RT‐PCR result. Most (155, 80.7%) specimens representing prolonged detection of SARS‐CoV‐2 RNA included in the analyses were collected from the upper respiratory tract, and of these, most were NP specimens, including NP/OP or NP/throat swab combinations (120, 62.2%) or assumed to be NP (30, 15.5%). Other respiratory specimens that tested positive for SARS‐CoV‐2 included nasal swabs (*n* = 1), throat or nose/throat (*n* = 4), sputum (*n* = 7), saliva (*n* = 2), OP (*n* = 4), pharyngeal swabs (*n* = 2), endotracheal/tracheal/bronchial swabs or secretions (*n* = 9), and BAL fluid (*n* = 11) (Table S1).

**FIGURE 2:**
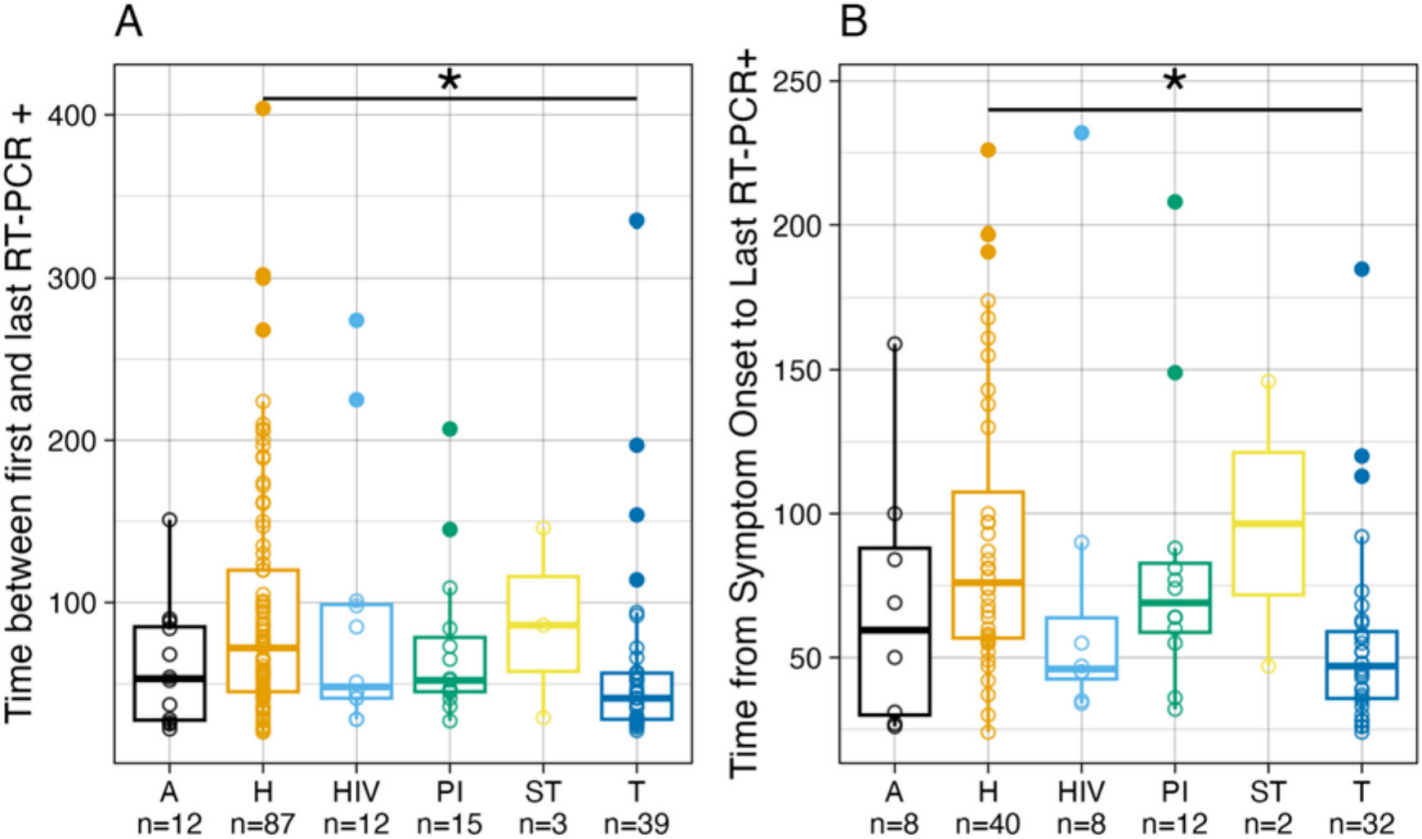
Duration of SARS‐CoV‐2 RNA in symptomatic immunocompromised patients from (A) time from the first to the last positive SARS‐CoV‐2 RT‐PCR result (*N* = 168) and (B) time from symptom onset to the last positive SARS‐CoV‐2 RT‐PCR result by primary immunocompromising condition (*N* = 102). *Indicates significant difference between groups with 95% confidence. A = autoimmune disease treatment. H = hematologic malignancy treatment. HIV = human immunodeficiency virus infection. PI = primary immunodeficiency. ST = solid tumor treatment. T = solid organ transplant.

Of the 175 symptomatic patients, 168 (96.0%) had information on time from the first to the last positive SARS‐CoV‐2 RT‐PCR result; the median duration of detectable SARS‐CoV‐2 RNA was 55.5 days, range 20–404 (IQR 41, 94.5). The median duration of detectable SARS‐CoV‐2 RNA in upper respiratory tract specimens (*n* = 147) from the first positive RT‐PCR result in symptomatic patients was 56 days, range 21–404 days (IQR 38.5, 99), and for lower respiratory tract specimens (*n* = 27), the median duration of SARS‐CoV‐2 RNA detection was 68 days, range 20–335 (IQR 45, 92.5). Among symptomatic patients, the duration from the first to the last detection of SARS‐CoV‐2 RNA varied by the following immunocompromising conditions: hematologic malignancy (median duration 72 days, *n* = 87), solid tumor malignancy (median duration 86 days, *n* = 3), patients receiving immunosuppressive treatment for an autoimmune disease (median duration of 53 days, *n* = 12), HIV infection (median duration of 48 days, *n* = 12), primary immunodeficiency (median duration of 52 days, *n* = 15), and solid organ transplant (median duration of 41 days; *n* = 39). However, the only statistically significant difference in median duration of detectable SARS‐CoV‐2 RNA from the first to the last positive RT‐PCR result among the immunocompromising diagnoses was that patients with hematological malignancies had significantly longer median duration of SARS‐CoV‐2 RNA detection than solid organ transplant recipients (adjusted *p =* 0.001302) (Figure 2A and Table 2).

**TABLE 2:** Underlying cause of immunocompromise and median days of SARS‐CoV‐2 RNA detection among symptomatic immunocompromised patients.

Immunocompromising condition	From onset of symptoms(*N* = 102)		From the first to the last RT‐PCR +(*N* = 168)	
	Median days (range)	Number of patients	Median days (range)	Number of patients
Autoimmune disease treatment	59.5 (26, 159)	8	53 (22, 151)	12
Hematologic malignancy treatment	76 (24, 226)	40	72 (20, 404)	87
Bone marrow transplant	74 (49, 87)	3	64.5 (22, 404)	10
CAR‐T therapy	74 (NA)	1	59 (23, 172)	5
Unspecified	79.5 (24, 226)	36	74.5 (20, 300)	72
HIV, by CD4+ cell count	46 (34, 232)	8	48 (28, 274)	12
< 50	72.5 (55, 90)	2	85 (45, 101)	5
50–200	45 (NA)	1	158 (41, 274)	2
> 200	45 (34, 232)	5	41 (28, 225)	5
Primary immunodeficiency	69 (32, 208)	12	52 (27, 207)	15
Solid tumor malignancy treatment	NA (47,146)	2	86 (29, 146)	3
Solid organ transplant treatment	47 (24, 185)	32	41 (21, 335)	39
Heart	42.5 (26, 120)	6	30.5 (23, 114)	8
Kidney	45 (24, 113)	19	42.5 (21, 154)	22
Liver	92 (48, 185)	3	72 (28, 335)	6
Lung	39.5 (28, 57)	4	28 (24, 57)	3

For 102 symptomatic patients with date of symptom onset, the median duration from onset to the last detection of SARS‐CoV‐2 RNA was 59 days, range 24–232 (IQR 45, 88). This included two patients with solid tumor malignancy (duration 47 and 146 days), 40 patients with hematologic malignancies (median duration 76 days), 8 patients receiving immunosuppressive treatment for an autoimmune disease (median duration 59.5 days), 12 patients with primary immunodeficiency (median duration 69 days), 8 patients with HIV infection (median duration 46 days), and 32 patients with solid organ transplant (median duration 47 days). However, the only statistically significant differences were observed in patients with hematological malignancies who had significantly longer median duration of SARS‐CoV‐2 RNA detection than solid organ transplant patients (adjusted *p =* 0.0011344) (Figure 2B and Table 2).

**FIGURE 3:**
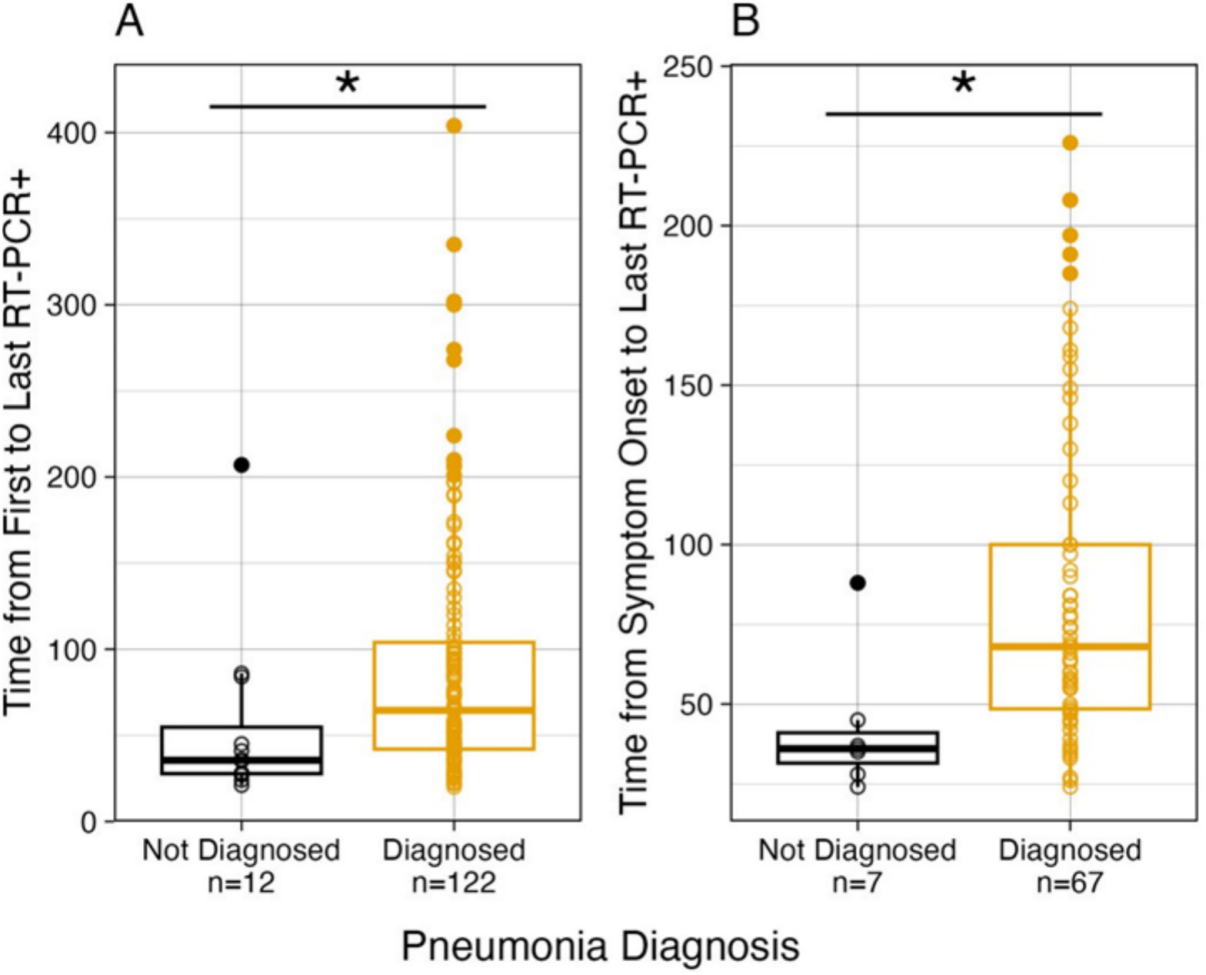
Duration of SARS‐CoV‐2 RNA in symptomatic immunocompromised patients with or without pneumonia diagnosis (*N* = 142; 122 with pneumonia; 20 without pneumonia). (A) Time from the first to the last positive SARS‐CoV‐2 RT‐PCR result (*N* = 134, 122 with pneumonia and 12 without pneumonia) and (B) time from symptom onset to the last positive SARS‐CoV‐2 RT‐PCR Result (*N* = 74; 67 with pneumonia; 7 without pneumonia).

There was a significant difference in the median duration of SARS‐CoV‐2 RNA detection from the first to the last positive RT‐PCR result between symptomatic patients with pneumonia (64.5 days, *n* = 122) and those without pneumonia (35.5 days, *n* = 12) (*p =* 0.01094) (Figure 3A). Similarly, there was a significant difference in the median duration of SARS‐CoV‐2 RNA detection from symptom onset to the last positive RT‐PCR result between symptomatic patients with pneumonia (68 days, *n* = 67) and without pneumonia (36 days, *n* = 7) (*p =* 0.004572, Figure 3B).

There was no statistically significant difference in the median duration of SARS‐CoV‐2 RNA detection from the first to the last positive RT‐PCR result between symptomatic patients who survived (57 days, *n* = 130) or died (61.5 days, *n* = 26) (*p =* 0.8157) or in the median duration of SARS‐CoV‐2 RNA detection from illness onset to the last positive RT‐PCR result among symptomatic patients who survived (59 days, *n* = 82) or died (71 days, *n* = 14) (*p =* 0.3176). Among symptomatic patients, no significant differences in the median duration from the first to the last positive RT‐PCR result or from illness onset to the last positive RT‐PCR result were observed in nonhospitalized versus hospitalized patients, among age groups, or by sex (Table S2).

Among eight asymptomatic patients, the median duration from the first to the last detection of SARS‐CoV‐2 RNA was 65.5 days (range 26‐142 days). The median duration from the first to the last detection of SARS‐CoV‐2 RNA was 47 days (range 26‐111) for six asymptomatic patients who were hospitalized for reasons other than COVID‐19 at the time of the first positive SARS‐CoV‐2 test and for two patients who were never hospitalized (durations of 105 and 142 days). There were no significant differences in duration of SARS‐CoV‐2 RNA detection by age group, sex, or hospitalization status (Table S3).

There was no significant difference in median duration of SARS‐CoV‐2 RNA detection from the first to the last positive RT‐PCR result between symptomatic (55.5 days, range 20–404, *n* = 168) and asymptomatic patients (65.5 days, range 26–142, *n* = 8) (*p =* 0.9405). Among all symptomatic and asymptomatic patients, there was no statistically significant difference in the median duration from the first to the last positive SARS‐CoV‐2 RNA results between hospitalized (55.5 days, *n* = 166) and nonhospitalized patients (124 days, *n* = 6) (*p =* 0.05179).

We performed a subset analysis on patients with viral genomic data and time between first and last SARS‐CoV‐2 RT‐PCR positive result (*n* = 47). The median duration of prolonged SARS‐CoV‐2 RNA detection was 109 days (IQR [70, 197]) with a maximum reported detection of 404 days [16]. For 23 patients with data on time since symptom onset, the median duration of prolonged SARS‐CoV‐2 detection was 100 days (IQR [68.5, 154]) with a maximum of 232 days [17]. There was no significant difference in the time between the first and last RT‐PCR positive result among patients with primary immunodeficiency (Figure S2, *p*‐value = 0.8226) nor for time since symptom onset (*p =* 0.9856).

### 3.5 Viral Culture

Among included patients, 50 (27.3%) patients from 39 publications had viral culture performed on respiratory specimens. Of these, 43 (86.0%) patients had one or more positive viral culture result (1 asymptomatic and 42 symptomatic patients). We focused on those respiratory specimens that represented the last timepoint from which virus was isolated. For 42 of these patients, only one specimen type was cultured at the final timepoint, whereas one patient had two specimen types that yielded recoverable virus at the final timepoint [18]. Virus was isolated mostly from upper respiratory specimens (39, 88.4%), including NP (*n* = 27) or NP assumed (*n* = 5), OP (*n* = 2), pharyngeal or throat swabs (*n* = 3), and NP/sputum (*n* = 1). Of the 43 patients with a positive viral culture for SARS‐CoV‐2, 27 had a hematologic malignancy, 9 were solid organ transplant recipients, 3 were receiving immunosuppressive treatment for an autoimmune disease, 2 had a primary immunodeficiency, and 1 had HIV infection.

All 43 patients with a positive viral culture had time reported since the first positive SARS‐CoV‐2 RT‐PCR result while 21 (48.8%) patients had reported time since symptom onset. The median duration from first detection of SARS‐CoV‐2 RNA to the last positive viral culture was 59 days, range 0–268 (IQR 24, 101). For 21 symptomatic patients with data on date of illness onset, the median time from symptom onset to the last positive viral culture was 60.5 days, range 0–238 (IQR 26, 100) (Figure S3). One asymptomatic patient (HIV patient with CD4 count < 50 cells/mm^3^) had SARS‐CoV‐2 isolated from a respiratory specimen collected 94 days after an initial SARS‐CoV‐2 RT‐PCR positive result.

## 4 Discussion

In this systematic review of published reports of SARS‐CoV‐2 RNA detection in respiratory specimens > 20 days in immunocompromised patients, the median duration from the first to the last positive SARS‐CoV‐2 RT‐PCR result was 57 days in upper respiratory and 65 days in lower respiratory tract specimens. The maximum duration of SARS‐CoV‐2 RNA detection in respiratory specimens was more than 400 days from the first positive SARS‐CoV‐2 RT‐PCR result with viral genomic data indicating prolonged SARS‐CoV‐2 RNA detection [16]. Among symptomatic patients with viral culture performed, the median duration of replication‐competent SARS‐CoV‐2 detection from symptom onset was 60.5 days and the longest duration of replication‐competent SARS‐CoV‐2 was 238 days since symptom onset. In symptomatic patients without date of onset reported, the longest duration of replication‐competent SARS‐CoV‐2 was 268 days from the first positive SARS‐CoV‐2 RT‐PCR result in an individual with untreated HIV. Our review suggests that immunocompromised patients hospitalized with pneumonia have prolonged SARS‐CoV‐2 infection compared to those without pneumonia. However, immunocompromised patients with severe pneumonia might be retested serially to inform isolation precautions and hospital discharge, whereas some patients with very mild symptoms or asymptomatic might not be tested for SARS‐CoV‐2 serially outside of participating in a study.

Our findings are similar to the results of recent studies of SARS‐CoV‐2 shedding in immunocompromised patients since emergence of the Omicron variant and subvariants. One prospective study published after our literature cutoff date reported that immunocompromised patients had detectable SARS‐CoV‐2 RNA for a median of 12 weeks, IQR 6‐31 weeks, with viable SARS‐CoV‐2 for a median of 4 weeks, IQR 3‐7 weeks [19]. Another recent prospective study reported that from the time of enrollment, 38 of 150 immunocompromised patients had evidence of SARS‐CoV‐2 RNA in respiratory specimens for more than 21 days, including 5 patients with SARS‐CoV‐2 RNA detected for 75–207 days and viable virus detected for 30–198 days [20].

Our review suggests that patients with hematologic malignancies can have longer duration of SARS‐CoV‐2 RNA and replication‐competent virus detection than other immunocompromised patients. Since our literature search cutoff date, additional studies in immunocompromised patients, including prospective studies, have reported data on prolonged SARS‐CoV‐2 RNA detection and or viral culture results. One recent prospective study reported that hematologic malignancy and transplant patients had significantly longer time to clearance of SARS‐CoV‐2 RNA and infectious virus from the upper respiratory tract compared to patients with autoimmunity or B‐cell deficiency [9]. Other studies have reported that hematologic malignancy requiring B‐cell depleting therapy is associated with prolonged SARS‐CoV‐2 shedding [19, 21]. A recent prospective study in immunocompromised patients with hematological malignancies receiving active chemotherapy or solid organ transplant recipients reported that duration of replication‐competent SARS‐CoV‐2 was significantly shorter in patients with higher neutralizing antibody levels against SARS‐CoV‐2 [22].

A key epidemiologic parameter for SARS‐CoV‐2 transmission and determining duration of transmission‐based precautions is the presence of symptoms. However, nearly half of the symptomatic patients included in our review had relapsing symptoms of COVID‐19 and detectable SARS‐CoV‐2 RNA in respiratory specimens during asymptomatic periods, and the median duration of SARS‐CoV‐2 RNA detection was similar in symptomatic and asymptomatic patients. In our review, two asymptomatic patients had recovery of infectious SARS‐CoV‐2 for at least 70 days, and longer detection of infectious SARS‐CoV‐2 in asymptomatic immunocompromised patients has been reported [23]. These findings highlight that the absence of symptoms may be a potentially unreliable indicator of infectiousness among immunocompromised patients with SARS‐CoV‐2 infection. In addition, there are reports of immunocompromised patients with COVID‐19 who have improved clinically with negative results of SARS‐CoV‐2 testing of upper respiratory tract specimens, who then had relapse of symptoms with clinical deterioration weeks to months later [24, 25], indicating that resolution of symptoms or even lack of detection of SARS‐CoV‐2 RNA in the upper respiratory tract of immunocompromised patients who were previously diagnosed with COVID‐19 does not exclude persistent SARS‐CoV‐2 infection.

Since prolonged detection of SARS‐CoV‐2 RNA does not necessarily inform whether infectious virus is present in the respiratory tract, and can overestimate the duration of infectious virus, viral culture results can help inform infection prevention and control measures and clinical management of immunocompromised patients with prolonged detection of SARS‐CoV‐2. The use of quantitative RT‐PCR assay results to inform whether infectious SARS‐CoV‐2 is present has been assessed by studies that correlated cycle threshold values with viral culture results for respiratory specimens. While detection of infectious SARS‐CoV‐2 has been correlated with low Ct values such as < 25 in some studies [26, 27], other studies have reported that SARS‐CoV‐2 could be isolated from respiratory specimens from a small number of patients with Ct values < 37 [28, 29, 30]. However, viral culture is not available for most patients due to requirements for specialized biosafety laboratory conditions not present at clinical laboratories. Sequencing of viral RNA in respiratory specimens was used to describe viral evolution and suggest prolonged SARS‐CoV‐2 replication for 335 days in a symptomatic immunocompromised patient [31]. Detection of subgenomic SARS‐CoV‐2 RNA has been used by some investigators as a surrogate marker to suggest the presence of replicating virus [32, 33], but most clinicians and patients do not have access to such laboratory methods outside of a research study. One study of SARS‐CoV‐2 infection in moderate to severely immunocompromised patients who received solid organ transplantation or chemotherapy against hematologic malignancy reported that SARS‐CoV‐2 rapid antigen testing had a negative predictive value of 92% for viable virus shedding in patients with symptom onset > 20 days [19].

### 4.1 Limitations

The findings of this review are subject to several limitations. First, due to resource limitations, our literature search was conducted as of July 1, 2022 and therefore did not include all published studies on detection of prolonged SARS‐CoV‐2 RNA or infectious virus shedding in immunocompromised patients after this date. However, as noted above, studies published after our literature search cutoff did not report longer detection of SARS‐CoV‐2 RNA or infectious virus. Second, because of publication bias, included patients did not represent all geographic regions or immunocompromised patient populations, limiting generalizability. Third, patients included in the analyses were identified from case reports and small retrospective case series without standardization of the collection of respiratory specimens, timing and intervals of sampling, specific SARS‐CoV‐2 RT‐PCR assays used, clinical and virologic data, antiviral treatments or monoclonal antibody products, or receipt of COVID‐19 vaccines, increasing variability. Fourth, the immunocompromised patients included in this review spanned a range of different SARS‐CoV‐2 variant periods, from the ancestral strain before COVID‐19 vaccines were available, and Alpha, Delta, and Omicron predominant periods, and our analyses were not able to assess differences in viral RNA or infectious virus detection due to limited virologic and genomic data. Fifth, although we excluded publications in which the authors reported that possible SARS‐CoV‐2 reinfection was present, most of the included patients did not have viral sequencing performed to assess whether reinfection with different SARS‐CoV‐2 variants had occurred. Therefore, it is possible that we might have misclassified some patients with reinfection as having prolonged duration of SARS‐CoV‐2 RNA. However, our analysis of a subset of 47 patients with available viral genomic data indicated that SARS‐CoV‐2 RNA was detectable for prolonged duration; these patients represented the maximum duration of SARS‐CoV‐2 RNA detection from the first to the last positive RT‐PCR result and from symptom onset to the last positive RT‐PCR result for all 183 included patients, increasing confidence with including all 183 patients in our analyses. Sixth, we were not able to assess and compare the effects of COVID‐19 vaccination, or antiviral or monoclonal antibody therapies on detection of viral RNA or infectious virus, as the availability of data on these and other therapies was lacking or incomplete for many patients, and some included patients were reported during the first pandemic wave before antiviral therapies were available. Seventh, the level of individual patient's degree of immunocompromise with respect to the timing of immunosuppressive medication regimens was not available. Regarding infectious virus detection, our inclusion criteria were not focused on viral culture data, and the subset of included patients with prolonged detection of replication‐competent SARS‐CoV‐2 may not be representative of all immunocompromised patients with viral culture data.

### 4.2 Public Health Implications

These observations have public health implications for COVID‐19 prevention and mitigation efforts, particularly in the identification of immunocompromised patient groups who may be at highest risk for persistent SARS‐CoV‐2 infection and potential transmission to close contacts. Immunocompromised patients, their close contacts, and caregivers should be informed about the risk of persistent SARS‐CoV‐2 infection and the importance of isolation. Our findings indicate that serial SARS‐CoV‐2 testing of respiratory tract specimens is important to inform infection prevention and control measures and duration of isolation for moderately or severely immunocompromised persons with SARS‐CoV‐2 infection, on a case‐by‐case basis. This is consistent with CDC guidance for a test‐based strategy to be used and (if available) consultation with an infectious disease specialist to determine when transmission‐based precautions can be discontinued for patients with SARS‐CoV‐2 infection who are moderately to severely immunocompromised [34]. In addition to resolution of fever without use of antipyretics and improvement of symptoms, both symptomatic and asymptomatic patients should continue to isolate until results are negative from at least two consecutive respiratory specimens collected 48 h apart (total of two negative specimens) tested using an antigen test or nucleic acid amplification test [34].

### 4.3 Conclusions

Immunocompromised persons can be potentially infectious with replication‐competent SARS‐CoV‐2 infection for prolonged periods due to long‐term persistent infection including while asymptomatic. Persons with hematologic malignancies or solid organ transplant recipients may be more likely to have prolonged detection of SARS‐CoV‐2 RNA and infectious virus. In addition to COVID‐19 vaccination and pre‐exposure prophylaxis with monoclonal antibody products if available, further prospective research studies and clinical trials are needed to identify optimal therapies and combinations of therapies to decrease the duration of SARS‐CoV‐2 shedding in immunocompromised patients.

## Author Contributions

**Rebecca C. Christofferson:** methodology, software, data curation, formal analysis, visualization, writing – review and editing, validation, investigation. **Jennifer E. Giovanni:** conceptualization, methodology, formal analysis, data curation, writing – original draft, investigation, writing – review and editing. **Emily H. Koumans:** investigation, writing – review and editing, data curation. **Muyiwa Ategbole:** investigation, data curation, writing – review and editing. **Samantha D. Clark:** investigation, data curation, validation, writing – review and editing. **Shana Godfred‐ Cato:** data curation, investigation, writing – review and editing. **Manoj P. Menon:** data curation, investigation, writing – review and editing. **Inka Sastalla:** data curation, investigation, writing – review and editing. **Beth K. Schweitzer:** data curation, investigation, writing – review and editing. **Timothy M. Uyeki:** conceptualization, methodology, investigation, validation, supervision, project administration, writing – review and editing.

## Ethics Statement

The authors have nothing to report.

## Consent

The authors have nothing to report.

## Conflicts of Interest

Shana Godfred‐Cato reports funding from HRSA for the Pediatric Pandemic Network to the University of Utah during completion of this study. All other authors declare no conflict of interest.

### Peer Review

The peer review history for this article is available at https://www.webofscience.com/api/gateway/wos/peer‐review/10.1111/irv.70121.

## Permission to Reproduce Material From Other Sources

Not applicable.

## Supporting information


**Figure S1** Classification of patients based on the confidence in confirming prolonged versus probable prolonged SARS‐CoV‐2 viral RNA detection and rationale.
**Figure S2:** Top) Distribution of time between first and last positive SARS‐CoV‐2 RT‐PCR results, or since symptom onset, among patients with viral genomic evidence of prolonged SARS‐CoV‐2 RNA duration (*n* = 47). Bottom) Distribution of duration of SARS‐CoV‐2 RNA detection according to primary immunodeficiency.
**Figure S3:** Duration of replication‐competent SARS‐CoV‐2 in viral culture for symptomatic patients by time since symptom onset (top panel A, *n* = 21) and by time from first positive SARS‐CoV‐2 RT‐PCR result (bottom panel B, *n* = 43) according to immunocompromising condition (*y*‐axis). Color of the dots represents detailed immunocompromising condition.
**Table S1:** Summary of the type of respiratory specimens represented at the time of last positive SARS‐CoV‐2 RT‐PCR result (*n* = 192). *Publication lists possible specimen types as: NP, sputum, or lower respiratory tract aspirates.
**Table S2:** Comparison of duration of SARS‐CoV‐2 RNA detection in symptomatic patients by demographic characteristics. Comparisons were tested using Kruskal–Wallis nonparametric test. Patients with “unknown” status for each characteristic were removed from analyses.
**Table S3:** Comparison of duration of SARS‐CoV‐2 RNA detection in asymptomatic patients from time from the first to the last positive SARS‐CoV‐2 result by demographic characteristics. Comparisons were tested using Kruskal–Wallis nonparametric test. Patients with “unknown” status for each characteristic were removed from analyses.

**Data S1** Supplementary Information.

## Data Availability

Data are provided as Supporting Information.
