# Correction to “A Systematic Review of Prolonged SARS‐CoV‐2 Shedding in Immunocompromised Persons”

**DOI:** 10.1111/irv.70127

**Published:** 2025-06-12

Christofferson, R. , Giovanni, J. , Koumans, E. , Ategbole, M. , Clark, S. , Godfred‐Cato, S. , Menon, M. , Sastalla, I. , Schweitzer, B. and Uyeki, T. (2025), A Systematic Review of Prolonged SARS‐CoV‐2 Shedding in Immunocompromised Persons. Influenza and Other Respiratory Viruses, 19: e70121, 10.1111/irv.70121.40394759 PMC12092234

The affiliations for Beth K. Schweitzer and Timothy M. Uyeki were listed incorrectly. The correct affiliation for both authors is “Centers for Disease Control and Prevention, Atlanta, Georgia, USA.”

We apologize for this error.

