# Supplementary material for: A Systematic Review of Prolonged SARS‐CoV‐2 Shedding in Immunocompromised Persons
Source: Influenza Other Respir Viruses. 2025 May 20;19(5):e70121. doi: 10.1111/irv.70121 (PMC12092234; doi:10.1111/irv.70121)
Supplement: Supplementary file 1 — Figure S1 Classification of patients based on the confidence in confirming prolonged versus probable prolonged SARS‐CoV‐2 viral RNA detection and rationale. Figure S2: Top) Distribution of time between first and last positive SARS‐CoV‐2 RT‐PCR results, or since symptom onset, among patients with viral genomic evidence of prolonged SARS‐CoV‐2 RNA duration (n = 47). Bottom) Distribution of duration of SARS‐CoV‐2 RNA detection according to primary immunodeficiency. Figure S3: Duration of replication‐competent SARS‐CoV‐2 in viral culture for symptomatic patients by time since symptom onset (top panel A, n = 21) and by time from first positive SARS‐CoV‐2 RT‐PCR result (bottom panel B, n = 43) according to immunocompromising condition (y‐axis). Color of the dots represents detailed immunocompromising condition. Table S1: Summary of the type of respiratory specimens represented at the time of last positive SARS‐CoV‐2 RT‐PCR result (n = 192). *Publication lists possible specimen types as: NP, sputum, or lower respiratory tract aspirates. Table S2: Comparison of duration of SARS‐CoV‐2 RNA detection in symptomatic patients by demographic characteristics. Comparisons were tested using Kruskal–Wallis nonparametric test. Patients with “unknown” status for each characteristic were removed from analyses. Table S3: Comparison of duration of SARS‐CoV‐2 RNA detection in asymptomatic patients from time from the first to the last positive SARS‐CoV‐2 result by demographic characteristics. Comparisons were tested using Kruskal–Wallis nonparametric test. Patients with “unknown” status for each characteristic were removed from analyses. [file IRV-19-e70121-s002.docx]

**Supplemental Information:**

Supplemental Text S1: Risk of Bias Assessment

S1.A Detailed Methodology. Two reviewers (RCC and SDC) used the JBI Checklist per the publication type (i.e. case report, case series, or cohort study) to assess publications for bias risk and for confidence in data synthesis. The checklist tool was applied to all included papers. For case reports, questions 1-4, if all data were provided, were answered “yes”. If some information was unclear, “unclear” was checked and justification for inclusion was provided in the comments. Questions 5-8 were marked as “not applicable” as the objective of the review did not include interventions, etc. For case series, if all relevant information was provided, questions 1-3 and 5-7 were answered “yes”. Consecutive inclusion of patients (Q4) and questions 8-10 were marked as “not applicable” for our study. Finally, for cohort studies, all but Q8 (concerning sufficient follow-up to capture necessary data) was checked as not applicable as the purpose was to abstract individual patient data and not to compare across cohort groups.

S1.B Data Synthesis Confidence. During the risk of bias assessment, fourteen included patients that had previously been deemed eligible were flagged for further review. Ten patients did not have enough data to sufficiently quantify the time between first and last positive SARS-CoV-2 RT-PCR result. Two patients did not have enough data to have confidence in classification as at least probable prolonged SARS-CoV-2 RNA detection, and one patient had six negative RT-PCR result between the first and last positive SARS-CoV-2 RT-PCR result. In 47 (25.7%) patients, genetic data was available to confirm prolonged SARS-CoV-2 RNA detection. In an additional 115 patients (62.8%), the timing of symptom resolution within the 90-day window or repeated SARS-CoV-2 RT-PCR testing was <90 days between consecutive positive results (Supplemental Figure S1). Further, we conducted additional analyses on only the patients with confirmed prolonged SARS-CoV-2 RNA detection, which we then used to justify our other criteria (see Results).

**Supplemental Figure S1:** Classification of patients based on the confidence in confirming prolonged versus probable prolonged SARS-CoV-2 viral RNA detection and rationale.

**
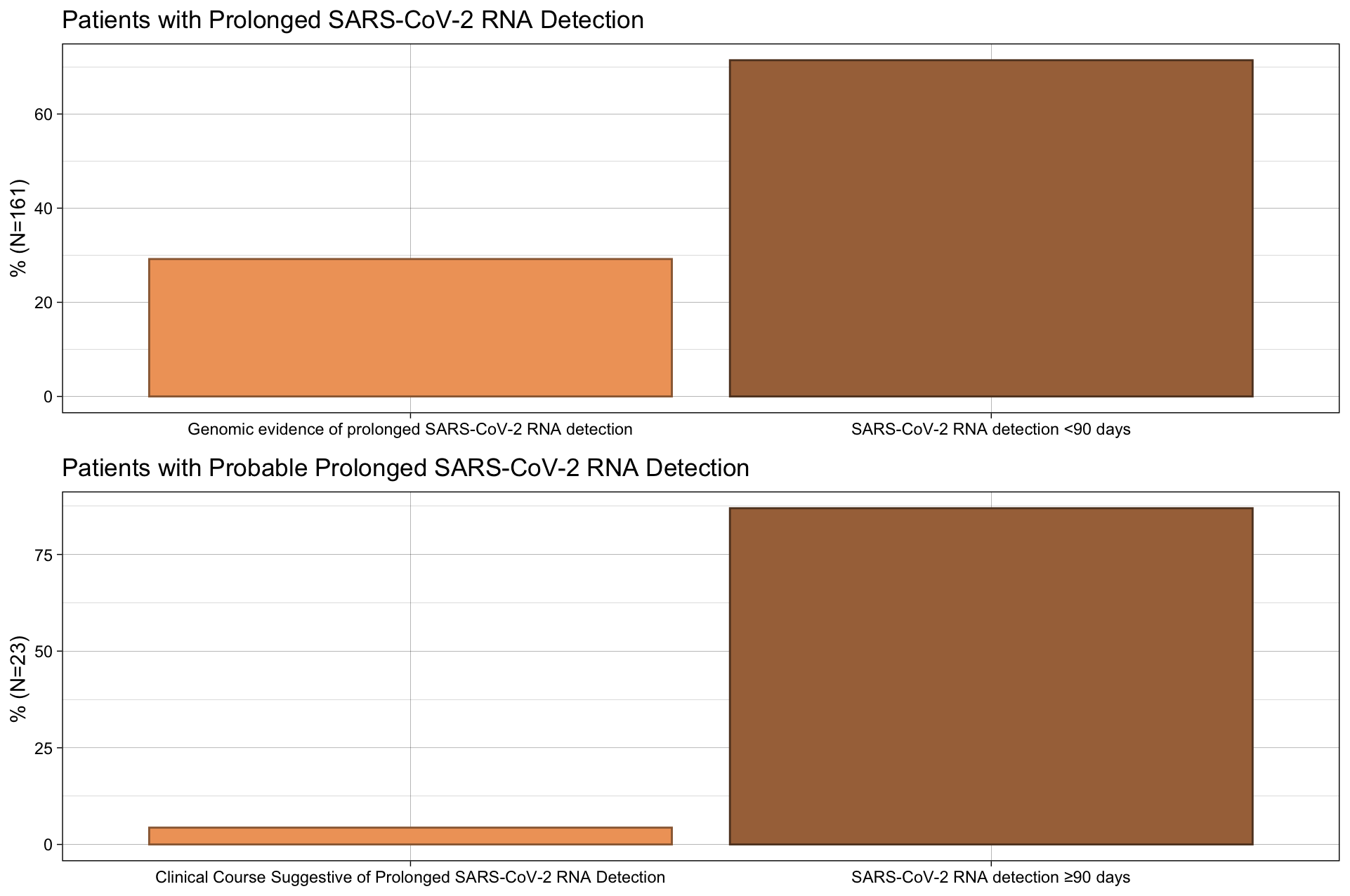
**

**
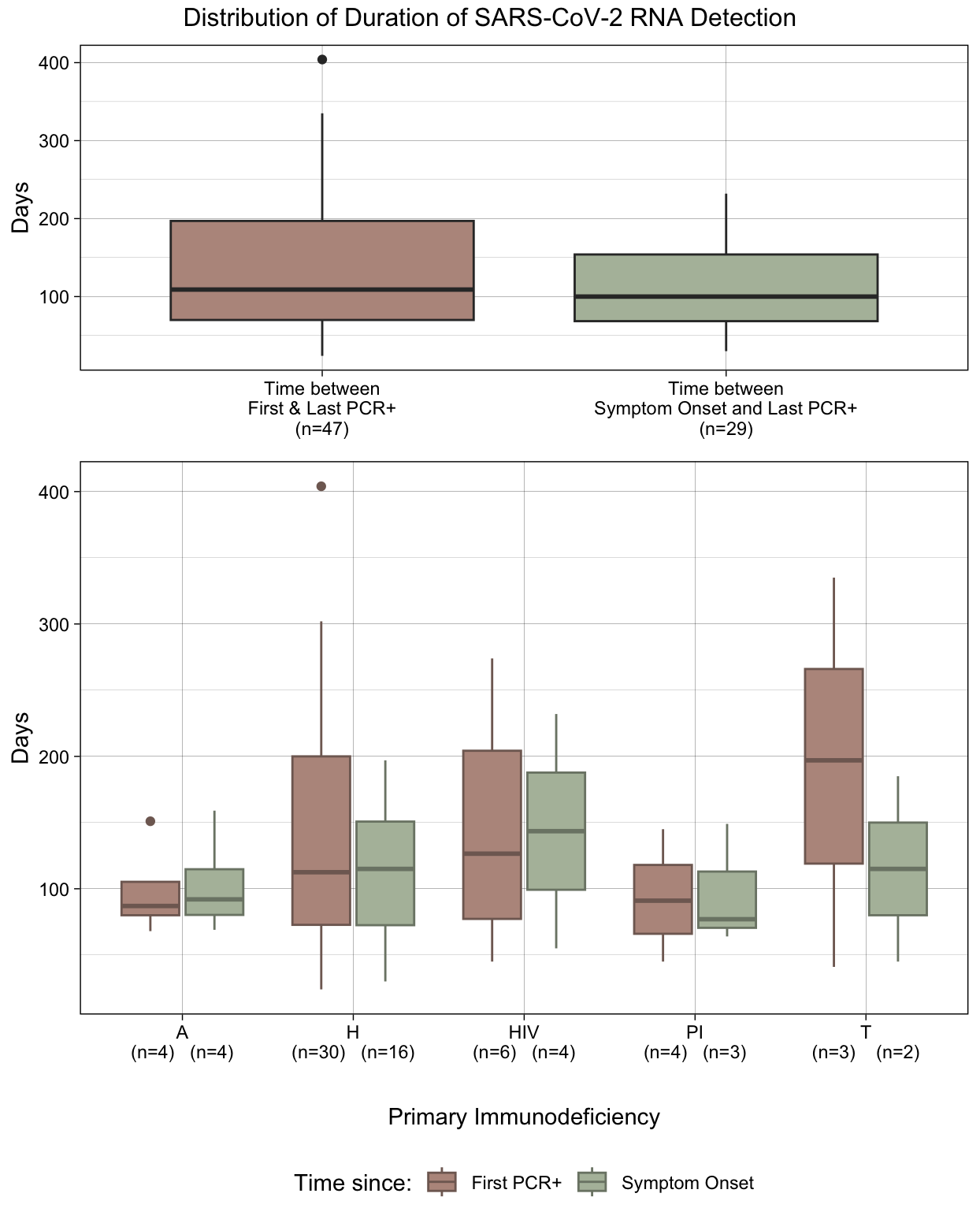
Supplemental Figure S2:** Top) Distribution of time between first and last positive SARS-CoV-2 RT-PCR results, or since symptom onset, among patients with viral genomic evidence of prolonged SARS-CoV-2 RNA duration (n=47). Bottom) Distribution of duration of SARS-CoV-2 RNA detection according to primary immunodeficiency.

**Supplemental Figure S3**: Duration of replication-competent SARS-CoV-2 in viral culture for symptomatic patients by time since symptom onset (top panel A, n=21) and by time from first positive SARS-CoV-2 RT-PCR result (bottom panel B, n=43) according to immunocompromising condition (y-axis). Color of the dots represents detailed immunocompromising condition.


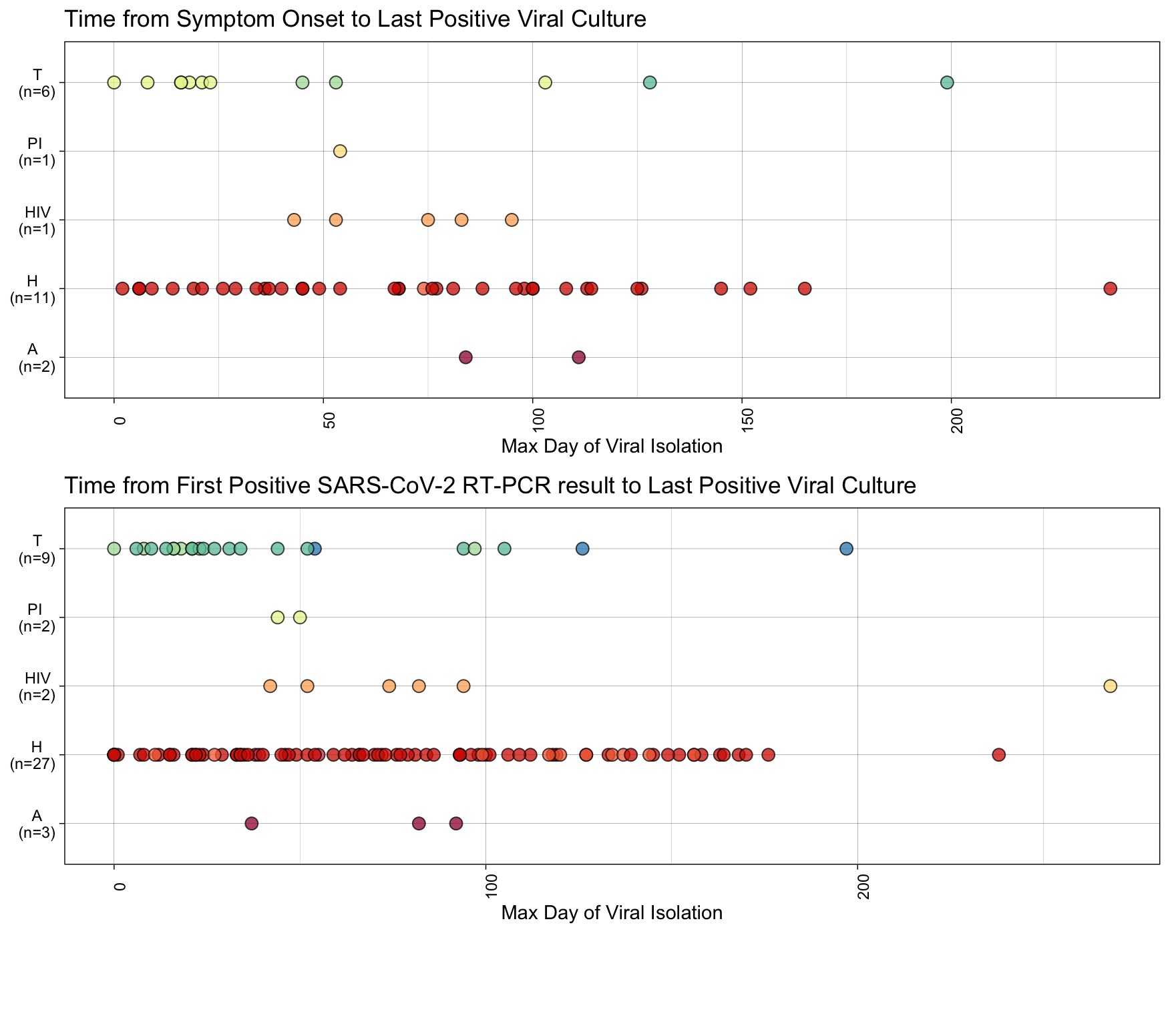


**Supplemental Table S1**: Summary of the type of respiratory specimens represented at the time of last positive SARS-CoV-2 RT-PCR result (n=192).

| **Sample Type** | **N** |
| --- | --- |
| BAL | 11 |
| Bronchial secretion | 7 |
| Endotrache al Aspirate | 1 |
| Nasal swab | 1 |
| Nose/Throat | 1 |
| NP | 110 |
| NP assumed | 29 |
| NP/OP | 9 |
| NP/Throat Swab | 1 |
| OP | 4 |
| Pharyngeal | 2 |
| Saliva | 2 |
| Sputum | 7 |
| Throat Swab | 3 |
| Tracheal sAspirate | 1 |
| Undetermined* | 4 |

**Publication lists possible specimen types as: NP, sputum, or lower respiratory tract aspirates.*

**Supplemental Table S2:** Comparison of duration of SARS-CoV-2 RNA detection in symptomatic patients by demographic characteristics. Comparisons were tested using Kruskal-Wallis non-parametric test. Patients with “unknown” status for each characteristic were removed from analyses.

| **Time Unit** | **Variable** (median days, n) | **p-value*** |
| --- | --- | --- |
| **Time from symptom onset to last positive SARS-CoV-2 RT-PCR result** | **Hospitalization**  Yes (58 days, n=98)  No (148 days, n=4) | 0.1651 |
|  | **Age Group**  <18 years (74 days, n = 5)  18-49 years (60 days, n=39)  50-64 years (56.5 days, n=34)  ≥65 years (60 days, n=22) | 0.7415 |
|  | **Sex**  Male (71 days, n=50)  Female (58 days, n=41) | 0.2657 |
|  | **Outcome**  Deceased (71 days, n=14)  Survived (59 days, n=82) | 0.3176 |
|  | **Pneumonia Diagnosis**  Yes (68 days, n=67)  No (36 days, n=7) | 0.004572 |
| **Time from first to last positive SARS-CoV-2 RT-PCR result** | **Hospitalization**  Yes (55.5 days, n=160)  No (146 days, n=4) | 0.2863 |
|  | **Age Group**  <18 years (56 days, n =10)  18-49 years (54 days, n=58)  50-64 years (53 days, n=61)  ≥65 years (68 days, n=34) | 0.8357 |
|  | **Sex**  Male (65 days, n=87)  Female (54 days, n=74) | 0.1973 |
|  | **Outcome**  Deceased (61.5 days, n=26)  Survived (57 days, n=130) | 0.8157 |
|  | **Pneumonia Diagnosis**  Yes (64.5 days, n=122)  No (35.5 days, n=12) | 0.01094 |

**Supplemental Table S3:** Comparison of duration of SARS-CoV-2 RNA detection in asymptomatic patients from time from first to last positive SARS-CoV-2 result by demographic characteristics. Comparisons were tested using Kruskal-Wallis non-parametric test. Patients with “unknown” status for each characteristic were removed from analyses.

| **Variable** | **p-value**** |
| --- | --- |
| **Hospitalization** (median days, n)  Yes (47 days, n=6)  No (124 days, n=2) | 0.09558 |
| **Age Group** (median days, n)  <18 years (27 days, n = 3)  18-49 years (29 days, n=3)  ≥65 years (108 days, n=2) | 0.1335 |
| **Sex** (median days, n)  Male (65.5 days, n=4)  Female (67 days, n=4) | 0.7728 |
